# Supplementary material for: Antileishmanial activity of synthetic analogs of the naturally occurring quinolone alkaloid N-methyl-8-methoxyflindersin
Source: PLoS One. 2020 Dec 28;15(12):e0243392. doi: 10.1371/journal.pone.0243392 (PMC7769561; doi:10.1371/journal.pone.0243392)
Supplement: Attachment — Submitted filename: Improving English letter.pdf [file pone.0243392.s001.pdf]

UNIVERSIDAD NACIONAL DE COLOMBIA
SEDE BOGOTÁ
FACULTAD DE CIENCIAS
DEPARTAMENTO DE FARMACIA
GRUPO DE INVESTIGACIÓN EN INMUNOTOXICOLOGÍA

Professor
Henk D. F. H. Schallig
Academic Editor
PLoS One

September 1st 2020

Dear Professor Schallig,

Following one of the reviewer's request regarding the English review of the manuscript entitled, **"Antileishmanial activity of synthetic analogues of the quinoline alkaloid N-methyl-8-methoxyflindersin"** by a native English-speaker, we hereby certify that the whole manuscript has been carefully reviewed by **Mr. Jason Garry**, a British translator that has been working with us at the Fundación Instituto de Inmunología de Colombia (FIDIC) for the last 24 years.

Yours sincerely,

Gabriela Delgado Murcia, DrSc
Corresponding author
Head of the Immunotoxicology Research Group
Faculty of Sciences
Universidad Nacional de Colombia

Manuel Alfonso Patarroyo MD, DrSc
Senior researcher
Head of the Molecular Biology and Immunology Department
Fundación Instituto de Inmunología de Colombia (FIDIC)

Carrera 30 No. 45-03, **FACULTAD DE CIENCIAS DEPARTAMENTO FARMACIA**, Edificio 450, 2º piso, Oficina 206
Teléfono: (57-1) 316 5120 Telefax: (57-1) 316 5060 Conmutador: (57-1) 316 5000 Ext. 14643 Fax: 14642
Correo electrónico: lgdelgadom@unal.edu.co
Bogotá, Colombia, Sur América
