# Supplementary material for: Antileishmanial activity of synthetic analogs of the naturally occurring quinolone alkaloid N-methyl-8-methoxyflindersin
Source: PLoS One. 2020 Dec 28;15(12):e0243392. doi: 10.1371/journal.pone.0243392 (PMC7769561; doi:10.1371/journal.pone.0243392)
Supplement: Attachment — Submitted filename: Response to reviewers V2.pdf [file pone.0243392.s002.pdf]

UNIVERSIDAD NACIONAL DE COLOMBIA
SEDE BOGOTÁ
FACULTAD DE CIENCIAS
DEPARTAMENTO DE FARMACIA
GRUPO DE INVESTIGACIÓN EN INMUNOTOXICOLOGÍA

Henk D. F. H. Schallig, Ph.D
Academic Editor
Plos One

November 18, 2020

Dear Dr Schallig,

Thank you for your kindly response about the process of submission of the manuscript entitled **"Antileishmanial activity of synthetic analogues of the naturally occurring quinolone alkaloid N-methyl-8-methoxyflindersin"** by Torres et al.

We have done the corrections indicated by the reviewer, and all the changes and the respective response on each point raised by the reviewer are exposed in the following table:

#	Reviewer observations	Answer
**General comments**		
1	This manuscript has improved with respect to structure and readability when compared to the first version. However, even though the authors have a native English speaker review the manuscript before resubmitting it, there are still some linguistic issues that must taken care of. The authors may consider asking a native English speaker for a second opinion.	The English of the article has been reviewed by a new official translator native to the language (Enago).
**Specific comments**		
TITLE		
2	Change title to: "Antileishmanial activity of synthetic analogues of the naturally occurring quinolone alkaloid N-methyl-8-methoxyflindersin"	The title of the article was changed from "Antileishmanial activity of synthetic analogues of the quinolone alkaloid N-methyl-8-methoxyflindersin" to

Carrera 30 No. 45-03, **FACULTAD DE CIENCIAS DEPARTAMENTO FARMACIA**, Edificio 450, 2º piso, Oficina 206
Teléfono: (57-1) 316 5120 Telefax: (57-1) 316 5060 Conmutador: (57-1) 316 5000 Ext. 14643 Fax: 14642
Correo electrónico: lgdelgadom@unal.edu.co
Bogotá, Colombia, Sur América

		"Antileishmanial activity of synthetic analogues of the naturally occurring quinolone alkaloid N-methyl-8-methoxyflindersin".
3	Change short title to: "Potential antileishmanial activity of quinoline alkaloids"	The short title of the article was changed from "Quinoline alkaloid and its potential antileishmanial activity" to "Potential antileishmanial activity of quinoline alkaloids"
ABSTRACT		
4	This section is okay, but the mistakes in lines 31-32 and 41-42 must be corrected<br>Indicate in lines 33-35 that these were in vitro studies.	Suggested corrections have made, and emphasis has made in In *vitro studies*
INTRODUCTION		
5	Lines 56-59. Make two separate sentences. Thus: "The first therapeutic choice for CL patients is based on the intravenous or intralesional administration of pentavalent antimony [refs]. Oral administration of miltefosine or intravenous administration of amphotericin B have also been recommended as second therapeutic possibilities [5,6]."	The change proposed has made. The references "Oliveira LF y cols. Acta Tropica. 2011", "Monge-Maillo B y cols. Drugs. 2013" y "Ponte-Sucre A y cols. PLoS Neglected Tropical Diseases. 2017", were added.
6	Lines 59-63. Make two separate sentences, improve the language, and clearer express the relationship between the statements in both sentences. Thus: "Nonetheless However, these drugs could produce adverse effects associated with such as cardiotoxicity, liver damage, nephrotoxicity, or even teratogenic effects in the case of miltefosine [7,8]. These drawbacks along with prolonged treatment schemes, and parenteral administration, can lead to non-compliance, and abandonment of prescribed treatment, and the consequent emergence of drug-resistant parasites could therefore occur [6,7,9]."	The change proposed has made.
7	Line 65. "….. and safer for patients (better adherence and less toxic effects) [10,11]." The authors must properly phrase this.	The changes were made by the paragraph: There is thus a need for new therapeutic alternatives that are more effective and efficient in terms of parasite elimination and disease resolution and safer for patients reflected in better adherence and less toxic effects there.

Carrera 30 No. 45-03, **FACULTAD DE CIENCIAS DEPARTAMENTO FARMACIA**, Edificio 450, 2º piso, Oficina 206
Teléfono: (57-1) 316 5080 Telefax: (57-1) 316 5060 Conmutador: (57-1) 316 5000 Ext. 14640 Fax: 14642
Correo electrónico: lgdelgadom@unal.edu.co
Bogotá, Colombia, Sur América

8	Lines 66-69. The authors must properly introduce the quinoline alkaloids by giving some relevant background information about these compounds.	The change has made. Relevant information was given related to the structure, as well as the activity of quinolinic alkaloid compounds, therefore the paragraph conforms to "Using natural molecules and their synthetic derivatives is the main strategy followed in the search for new therapeutic options [7]. Of these, quinoline alkaloids, which are secondary metabolites found mainly in plants from the Rutaceae family, are biosynthesized from anthranilic acid and comprise carboxyl groups of anthranilic acid with an acetate group (malonate) and their subsequent cyclization of the quinolinic ring [12,13]. [12,13]. Quinoline alkaloids are effective against CL caused by L. amazonensis and L. venezuelensis in BALB/c mice [14]. In addition, 2-substituted quinoline alkaloids chimanine D and B isolated from the Galipea longiflora K. Krause stem bark act against L. braziliensis and L. donovani promastigotes [15,16]."
9	Line 80. The authors should connect this alinea with the previous one by using, for instance, the expressions "Therefore, ……" or For this reason,"……..".	The change proposed has made. Therefore the paragraph is set to "For this reason, in silico tools ...".
MATERIALS AND METHODS		
10	The authors may consider substituting the title of the first subsection by "Screening for synthetic analogues of N-methyl-8-methoxyflindersine"	The change proposed has made, therefore the title changes from "Screening for synthetic compounds analogous to the natural quinoline alkaloid" to " Screening for synthetic analogs of compound 1".
11	The authors may consider combining the "subsection "Isolating human monocyte-derived macrophages" with the subsection "Antileishmanial activity in L. (V.) panamensis intracellular amastigotes"	It is considered not to make changes in the order of the sub-sections, to make it easier to read separately, the requirements of cell culture and each technical apart; because they all share the same cellular model (hMDMs). But we change the title to "Isolating hMDMs".

Carrera 30 No. 45-03, **FACULTAD DE CIENCIAS DEPARTAMENTO FARMACIA**, Edificio 450, 2º piso, Oficina 206
Teléfono: (57-1) 316 5080 Telefax: (57-1) 316 5060 Conmutador: (57-1) 316 5000 Ext. 14640 Fax: 14642
Correo electrónico: lgdelgadom@unal.edu.co
Bogotá, Colombia, Sur América

12	The authors may consider placing the subsections “Inducing cell death in human monocyte-derived macrophages”, “Evaluating reactive oxygen species (ROS) and nitric oxide (NO) production in hMDM” (no abbreviations in section title), and “Transmission electron microscopy of *L. (V) panamensis* infected macrophages” immediately behind the subsection “Antileishmanial activity in *L. (V.) panamensis* intracellular amastigotes”	We make the changes of place of the suggested sections: "Inducing apoptosis in hMDMs", "Evaluating reactive oxygen species (ROS) and nitric oxide (NO) production in hMDMs" (no abbreviations in section title), and "Transmission electron microscopy of *L. (V) panamensis* infected hMDMs" immediately behind the subsection "Antileishmanial activity in *L. (V.) panamensis* intracellular amastigotes"
13	The authors may consider placing the “Ethics statement” to the very end of the “Materials and Methods section	The change proposed has made, therefore that subsection was moved by the end of the Methods section.
14	The authors may consider placing the data processing of each methodology under the corresponding subsection and rewrite the subsection “Data processing and statistical analysis” to a subsection “Statistical analysis”.	The change proposed has made, therefore the title changed from "Data processing and statistical analysis" to subsection "Statistical analysis".
RESULTS		
15	The authors may consider thinking up better titles for the subsections. For instance, “In silico studies” may be substituted by “Synthetic analogues of N-methyl-8-methoxyflindersine”	Replaced with "Synthetic analogues of compound 1"
16	Similarly, the authors may come up with better titles than “In vitro cytotoxic and antileishmanial activity”, “Evaluating antileishmanial activity and safety in vivo”, etc.	The changes suggested has made. The titles were replaced: “In vitro evaluation of antileishmanial activity”, “Biochemical (Nitric Oxide and Reactive Oxygen Species production) and structural changes in infected hMDMs”, “Induction of cell apoptosis in *L. (V.) panamensis*- infected hMDMs”, “*L. (V.) panamensis*-infected hMDM ultrastructural alterations”, “Ultrastructural modifications of quinoline alkaloid-like compounds in *L. (V.) panamensis* intracellular amastigotes”
DISCUSSION		
17	The authors must properly connect the second alinea to the first. Just mentioning that “Quinoline alkaloids have been tested for their strong antiparasitic properties	The change suggested by the reviewer has made. Besides, the emphasis has made on the relevance of the compound N-

Carrera 30 No. 45-03, **FACULTAD DE CIENCIAS DEPARTAMENTO FARMACIA**, Edificio 450, 2º piso, Oficina 206
Teléfono: (57-1) 316 5080 Telefax: (57-1) 316 5060 Conmutador: (57-1) 316 5000 Ext. 14640 Fax: 14642
Correo electrónico: lgdelgadom@unal.edu.co
Bogotá, Colombia, Sur América

	[13,14,33]"(line 449). For instance, the authors may take their time and mention that quinoline alkaloids including N-methyl-8-methoxyflindersine, have shown antileishmanial activity in vitro, and that synthetic analogues of N-methyl-8-methoxyflindersine have been tested in the current study. Etc., etc. The authors should take their time explaining tjeri train of though, obviosuly, without exaggerating.	methyl-8-methoxyflindersine as antileishmanial, and the *In vitro* evaluation of synthetic compounds.
REFERENCES		
18	The authors must make sure that all references in their reference list are according to the format of PLOS One. That is, with the journal names properly abbreviated.	The change suggested by the reviewer has made to the references: 4,7,9,10,12,13,15,20,24,28,29,33,34,36,38,39,41 and 47 (The journal names, were properly abbreviated).

In addition to the above, we also want to inform that the paper has been edited for English language, grammar, punctuation, and spelling by organization specialized in this type of service (Enago),

We look forward to hearing from you,

Yours sincerely

Gabriela Delgado Murcia, DrSc
Corresponding author
Head of the Immunotoxicology Research Group
Faculty of Sciences
Universidad Nacional de Colombia

Carrera 30 No. 45-03, **FACULTAD DE CIENCIAS DEPARTAMENTO FARMACIA**, Edificio 450, 2º piso, Oficina 206
Teléfono: (57-1) 316 5080 Telefax: (57-1) 316 5060 Conmutador: (57-1) 316 5000 Ext. 14640 Fax: 14642
Correo electrónico: lgdelgadom@unal.edu.co
Bogotá, Colombia, Sur América
